# Phenotypic and genotypic characterization of linezolid resistance and the effect of antibiotic combinations on methicillin-resistant *Staphylococcus aureus* clinical isolates

**DOI:** 10.1186/s12941-023-00574-2

**Published:** 2023-04-03

**Authors:** Asmaa I. AbdAlhafiz, Nooran S. Elleboudy, Khaled M. Aboshanab, Mohammad M. Aboulwafa, Nadia A. Hassouna

**Affiliations:** 1 Department of Microbiology and Immunology, Faculty of Pharmacy, Ain Shams University, Cairo, Egypt; 2 Faculty of Pharmacy, King Salman International University, South Sinai, Ras-Sudr, Egypt

**Keywords:** MRSA, Linezolid, Biofilm, 23 S rRNA, Ribosomal genes, Mutations, *Cfr(b)* gene, Antibiotic combinations, Synergism

## Abstract

**Background:**

Methicillin-Resistant *Staphylococcus aureus* (MRSA) causes life-threatening infections, with narrow therapeutic options including: vancomycin and linezolid. Accordingly, this study aimed to characterize phenotypically and genotypically, the most relevant means of linezolid resistance among some MRSA clinical isolates.

**Methods:**

A total of 159 methicillin-resistant clinical isolates were collected, of which 146 were indentified microscopically and biochemically as MRSA. Both biofilm formation and efflux pump activity were assessed for linezolid-resistant MRSA (LR-MRSA) using the microtiter plate and carbonyl cyanide 3-chlorophenylhydrazone (CCCP) methods, respectively. Linezolid resistance was further characterized by polymerase chain reaction (PCR) amplification and sequencing of domain V of 23 S rRNA; *rplC*; *rplD*;and *rplV* genes. Meanwhile, some resistance genes were investigated: *cfr; cfr(B); optrA; msrA;mecA;* and *vanA* genes. To combat LR-MRSA, the effect of combining linezolid with each of 6 different antimicrobials was investigated using the checkerboard assay.

**Results:**

Out of the collected MRSA isolates (n = 146), 5.48% (n = 8) were LR-MRSA and 18.49% (n = 27) were vancomycin-resistant (VRSA). It is worth noting that all LR-MRSA isolates were also vancomycin-resistant. All LR-MRSA isolates were biofilm producers (r = 0.915, *p* = 0.001), while efflux pumps upregulation showed no significant contribution to development of resistance (t = 1.374, *p* = 0.212). Both *mecA* and *vanA* genes were detected in 92.45% (n = 147) and 6.92% (n = 11) of methicillin-resistant isolates, respectively. In LR-MRSA isolates, some 23 S rRNA domain V mutations were observed: A2338T and C2610G (in 5 isolates); T2504C and G2528C (in 2 isolates); and G2576T (in 1 isolate). Amino acids substitutions were detected: in L3 protein (*rplC* gene) of (3 isolates) and in L4 protein (*rplD* gene) of (4 isolates). In addition,* cfr(B)* gene was detected (in 3 isolates). In 5 isolates, synergism was recorded when linezolid was combined with chloramphenicol, erythromycin, or ciprofloxacin. Reversal of linezolid resistance was observed in some LR-MRSA isolates when linezolid was combined with gentamicin or vancomycin.

**Conclusions:**

LR-MRSA biofilm producers’ phenotypes evolved in the clinical settings in Egypt. Various antibiotic combinations with linezolid were evaluated in vitro and showed synergistic effects.

**Supplementary Information:**

The online version contains supplementary material available at 10.1186/s12941-023-00574-2.

## Introduction

Every year about 20,000 deaths are reported due to MRSA infections in the United States (U.S.) alone [1, 2]. In addition to resistance, MRSA possesses a vast capacity for adopting various virulence factors, including both biofilm formation and production of: toxins; adhesins; enzymes; or immunomodulators [3, 4]. MRSA causes a diversity of infections as skin and soft tissue, wound infections, osteomyelitis, infective endocarditis, deep tissue abscesses, and hospital-acquired (HAP) and ventilator-associated pneumonia (VAP) which may lead to fatal bacteremia and sepsis [1, 2, 5]. Unfortunately, very few last resort antimicrobials can be used to treat such serious infections, namely: ceftaroline; daptomycin; linezolid; teicoplanin; and vancomycin [6].

Linezolid is a completely synthetic antimicrobial agent, considered the leading member of the oxazolidinone class. Since the year 2000, it has been Food and Drug Administration (FDA) approved for clinical use in the U.S. against severe Gram-positive infections as MRSA, methicillin-resistant coagulase-negative Staphylococci (MRCoNS), and VRE infections [6, 7]. Linezolid inhibits protein synthesis through hindering initiation of 70 S protein complex by specifically fitting at site P of the peptidyl transferase center (PTC) surrounded by domain V of 50 S ribosomal subunit of 23 S rRNA, thus interfering with aminoacyl tRNA addition on site A [6–9].

Linezolid resistance was first reported in Staphylococci in 2001, 1 year after its approval by FDA in the U.S [6]. Luckily, linezolid resistance is not very frequent in Staphylococci [10–13]. Viñuela-Prieto et al*.* reported that linezolid resistance is still clinically scarce among MRSA isolates, and usually linked to nosocomial outbreaks [14]. Acquirement of linezolid resistance has been linked to both previous exposure and duration of therapy [10, 15].

Gram-positive bacteria possess two main strategies to acquire linezolid resistance: mutational and non-mutational mechanisms [6]. Spontaneous de novo mutations have been reported in: i. domain V of 23 S rRNA gene copies as G2576T, T2500A and G2447T; ii. some genes as *rplC, rplD* and *rplV* encoding ribosomal proteins L3, L4, and L22, respectively [6]. For non-mutational mechanisms, some genes have been detected in linezolid resistant isolates as *cfr*, *cfr(B)*, *optrA*, and *msrA* genes [6, 8, 9, 16]. Additionally, other mechanisms as biofilm formation and efflux pump expression have sometimes been reported to have an impact on the minimum inhibitory concentrations (MICs) of different antimicrobials including linezolid [17, 18].

The mutational mechanisms attributed to linezolid resistance are most often a consequence of prior linezolid treatment, and cannot disseminate [6, 10]. The non-mutational mechanisms causing linezolid resistance do not demand previous exposure in most cases and are usually mediated by gene transfer among clinical isolates [12]. Both the *cfr* and *optrA* genes are the most reported in linezolid resistant *S. aureus* (LRSA), and LR-MRSA clinical isolates [9, 13]. The *cfr* gene is the chloramphenicol-florfenicol resistance gene, it may be related to the phenicol, lincosamide, oxazolidinone, pleuromutilin, and streptogramin A (PhLOPSA resistance phenotype) resistance as all of them exert their action by binding in ribosomal PTC [9, 19]. The *cfr* gene is a mobile gene either harbored on a plasmid or located in unstable chromosomal region, so it is commonly responsible for linezolid resistance outbreaks [11, 14]. The *cfr(B)* gene has been reported in linezolid resistant Gram-positive isolates, it is a *cfr*-like gene with 75% amino acid similarity to cfr protein usually detected in Staphylococci and Enterococci [19]. Moreover, the *optrA* gene has been as well reported to be linked to linezolid resistance in Gram-positive clinical isolates such as* E. faecalis* and *E. faecium*. It mediates expression of ATP binding cassette-F (ABC-F) transporters, is plasmid mediated and may be related to phenicols and oxazolidinones resistance [6, 9, 11, 20]. In the same context, *Staphylococcal msrA* gene is responsible for MsrA protein expression which imparts inducible resistance to macrolides by actively pumping out antimicrobial molecule [16, 21].

Using antibiotic combinations is a very common and promising strategy to face resistance, as these combinations result in resistance reduction and enhancement of efficacy [22]. Some studies reported that combination therapy is an auspicious strategy for treatment of MRSA infections [22–24]. The i*n vitro* activity of different antibiotic combinations was investigated especially against MRSA infections with high rate of vancomycin therapeutic failure [25]. Linezolid combinations with different antimicrobials (daptomycin, gentamicin, erythromycin, tetracycline, imipenem, and plazomicin) were in vitro investigated, where variable combinatory effects were reported against MRSA isolates [22, 24, 25]. Accordingly, the aim of the present study was to investigate the phenotypic characteristics and the molecular basis of linezolid resistance among LR-MRSA clinical isolates recovered from a major tertiary hospital in Cairo, Egypt, and to investigate the possible in vitro synergistic effects of linezolid combinations with different antimicrobials against LR-MRSA clinical isolates.

## Methods

### Bacterial isolates

A total of 146 MRSA were collected from El-Demerdash Hospital’s microbiology laboratory, Cairo, Egypt, from December 2020 to August 2021. MRSA isolates were recovered from different clinical specimens, including: wound exudates (45.89%); sputum and bronchial aspirates (39.73%); and blood cultures (14.38%).

### Identification of MRSA isolates

Identification was initially carried out microscopically by Gram staining and by culturing on mannitol salt agar (MSA, HiMedia, India), and *Staphylococcus* medium 110 (Difco, Japan). Then biochemically by catalase and coagulase tests [26, 27], followed by API^®^ identification kit which was used according to the manufacturer’s instructions (BioMérieux, France).

Methicillin resistance was detected as specified by the guidelines of Clinical and Laboratory Standards Institute (CLSI) [28] using cefoxitin (30 µg/disk; HiMedia, India) disk diffusion assay. An isolate is considered methicillin resistant when inhibition zone diameter (IZ) ≤ 21 mm [28]. Reference strains methicillin sensitive *S. aureus* (MSSA) ATCC 25923 and MRSA ATCC 43300 were used as negative and positive controls, respectively.

### Antimicrobial susceptibility testing

#### Antibiogram analysis

The antibiogram analysis was conducted according to CLSI guidelines [28] using the commercially available discs (HiMedia, India) of 16 antimicrobial agents belonged to 10 different classes. The tested antibiotics were: amoxicillin/clavulanic (20/10 µg/disk, penicillins); ceftriaxone (30 µg/disk, cephalosporins); cefepime (30 µg/disk, cephalosporins); chloramphenicol (30 µg/disk); ciprofloxacin (5 µg/disk, fluoroquinolones); clindamycin (2 µg/disk, macrolides); doxycycline (30 µg/disk, tetracyclines); erythromycin (15 µg/disk, macrolides); gentamicin (10 µg/disk, aminoglycosides); levofloxacin (5 µg/disk, fluoroquinolones); linezolid (30 µg/disk, oxazolidinone); moxifloxacin (5 µg/disk, fluoroquinolones); oxacillin (5 µg/disk, penicillins); tigecycline (15 µg/disk, tetracyclines-derived glycylcycline); trimethoprim/sulfamethoxazole (1.25/23.75 µg/disk, sulphonamides); and vancomycin (30 µg/disk, glycopeptide).

A bacterial suspension of each isolate was freshly prepared, then bacterial count was adjusted to 1×10^8^ colony-forming units (CFU)/mL using 0.5 McFarland standard. Mueller Hinton agar plates (MHA, HiMedia, India) were inoculated using sterile swabs by surface streaking in three different directions. The plates were incubated at 37 ℃ for 16–18 h, except coagulase negative (CoNS) plates that were incubated for 24. Reference strain *S. aureus* ATCC^®^ 29213 was used for quality control for linezolid and vancomycin resistance. The results were interpreted according to the CLSI guidelines [28].

#### Determination of MIC

The MICs were determined using broth microdilution (BMD) method for some antibiotics, namely: chloramphenicol (Orchidia Pharmaceutical Industries, Egypt); ciprofloxacin (Amriya Pharmaceutical Industries, Egypt); erythromycin (Sigma-Aldrich Co., Germany); gentamicin (Memphis Co. for Pharmaceuticals & Chemical Industries, Egypt); linezolid (Averroes pharma for pharmaceutical industries, Egypt); tigecycline (Pfizer Inc, Philadelphia, U.S.); and vancomycin (Lyomark Pharma Co., Germany).

The MICs were determined according to the CLSI guidelines [29] using BMD method. Two-fold serial dilutions in cation-adjusted Mueller Hinton broth (caMHB, HiMedia, India) were prepared from the antimicrobial stock solutions (1024 µg/mL) and dispensed in all columns of the 96-well microtiter U-shaped bottom plates except column 11 and 12 as they were used as positive and negative controls. Inocula (10 µL each) of microbial count adjusted to 1×10^6^ CFU/mL were transferred into all wells except the negative control. The final count was 1×10^5^ CFU/mL. Microtiter plates were incubated at 37 ℃ for 16–20 h for all tested antimicrobial agents except that for 24 h in case of vancomycin. A reference strain *S. aureus* ATCC^®^ 29213 was used for quality control. The obtained results were interpreted according to clinical breakpoints presented by CLSI, M07-A10 protocol, and breakpoint tables version 10.0, 2020 of the European Committee on Antimicrobial Susceptibility Testing (EUCAST) for the tigecycline [29, 30].

### Phenotypic investigation of resistance mechanisms in the LR-MRSA isolates

#### Investigating the role of efflux pumps in linezolid resistance

This was done using CCCP (Sigma-Aldrich, USA). Firstly, MICs of CCCP were determined, then MICs of linezolid in presence of 0.5 MIC of CCCP were determined [31–33]. The MICs were determined by BMD method according to the CLSI guidelines as mentioned earlier [28]. The reference *E. coli* ATCC^®^ 25922 strain was used for quality control of CCCP MIC [34, 35]. An eight-fold or more reduction in the MIC values indicates a significant contribution of efflux pumps to the antimicrobial resistance [33, 35].

#### Investigating biofilm formation

The biofilm formation ability of the LR-MRSA isolates were investigated using the crystal violet staining in microtiter plates according to [36], and [37] with slight modification. About 5 mL of tryptic soy broth (TSB) supplemented with 1% glucose (Tryptone 1.7% (Qualikems Fine Chem Pvt. Ltd, India), Soy 0.3% (LabM, UK), NaCl 0.5% and K_2_HPO_4_ 0.25%, and 1% glucose (El Nasr Pharmaceutical Chemicals Co. (ADWIC), Egypt)), were inoculated with a loopful of each isolate and incubated at 37 ℃ for 18–20 h. After incubation, about 200 µL aliquots of bacterial suspensions (count adjusted to 1×10^6^ CFU/mL) were transferred into wells of 96-well flat-bottom microtiter plates. Three independent biofilm formation quantification were done, meanwhile eight replicates were done for each isolate. Plates were incubated at 37 ℃ for 48 h then decanted, and washed 3 times using sterile phosphate buffered saline (PBS, pH = 7.2). Hot air drying at 60 ℃ for 1 h was applied for fixation of the preformed biofilms. The adherent biofilms were stained by 0.1% crystal violet (Alpha Chemika, India) at room temperature for 15 min. The crystal violet was aspirated gently and plates were washed with sterile distilled water. The intensity of the preformed biofilm was measured spectrophotometrically at A_550_ after adding 33% glacial acetic acid [El Nasr Pharmaceutical Chemicals Co. (ADWIC), Egypt] [36, 37]. Categorization was done by determination of the cut-off value of negative control (ODc), where ODc equals the average of the uninoculated broth optical densities (OD) added to 3 standard deviation: (i) OD ≤ ODc no biofilm production, (ii) ODc < OD ≤ 2 × ODc weak biofilm production, (iii) 2 × ODc < OD ≤ 4 × ODc moderate biofilm production, and (iv) 4 × ODc < OD strong biofilm production [38]. The *S. aureus* ATCC^®^ 43300 was used as a positive control and uninoculated medium was used as a negative control.

### Genotypic characterization of linezolid resistance

#### Plasmid extraction and detection

The plasmid extraction was done using the GeneJet^®^ Plasmid Miniprep kit (ThermoScientific, U.S), according to the manufacturer’s instructions. The plasmid detection and analysis were done using 1% agarose gel electrophoresis, in presence of 1 kilobase pair (Kbp) DNA Ladder ready to use (RTU, GeneDirx, Taiwan) [39].

#### Molecular detection of linezolid resistance genes

The chromosomal DNA was extracted using GeneJet^®^ genomic DNA purification kit (ThermoScientific, U.S) according to the manufacturer’s instructions. The Domain V of the 23 S ribosomal RNA, *rplC, rplD,* and *rplV* genes, and some other genes;* cfr, cfr(B), icaA, mecA, msrA, optrA,* and* vanA*, were amplified using conventional PCR using thermocycler (Techne TC-412^™^, UK). The primers used (Table 1) were synthesized by Macrogen, Korea. PCR mixture (50 µL) was prepared using 25 µL MyTaq^™^ Red Mix PCR (2X) master mix (Bioline, Germany), 1 µL forward primer P_f_ (20 pmol/μL), 1 µL reverse primer P_r_ (20 pmol/μL), 2 µL DNA extract, and 21 µL nuclease-free water (ThermoFisher Scientific, USA). Amplification was conducted by initial denaturation at 95 ℃ for 4 min; 30 cycles of denaturation at 95 ℃ for 30 s, (annealing temperature listed in Table 1) for 45 s, elongation at 72 ℃ for 1 min, then a final elongation at 72 ℃ for 10 min [9, 40–45]. PCR products were separated on 1.2% agarose gel (Fisher scientific, US) using 100 base pair (bp) DNA ladder (cleaverscientific, UK), and 1 Kb DNA Ladder ready to be used (RTU, GeneDirx, Taiwan) [39]. The reference strains *S. aureus* ATCC 25923 and *S. aureus* ATCC 43300 were used for quality control [28].

**Table 1:** Tabular illustration of the used primers sequence, annealing temperatures (Ta), expected product sizes (bp), and their references

Gene name	Primer name	Primer sequence (5′-3′)	Annealing temperature Ta (ºC)	Product size (bp)	Reference
*mecA*	*mecA*-F	AAAATCGATGGTAAAGGTTGGC	53	533	[47]
	*mecA*- R	AGTTCTGGAGTACCGGATTTGC			
*icaA*	*icaA*-F	GAC CTC GAA GTC AAT AGA GGT	60	814	[45]
	*icaA*-R	CCC AGT ATA ACG TTG GAT ACC			
Domain V of *23S rRNA*	Domain V of *23S rRNA*-F	GCGGTCGCCTCCTAAAAG	55	390	[9]
	Domain V of *23S rRNA*-R	ATCCCGGTCCTCTCGTACTA			
*rplC*	*rplC*-F	AACCTGATTTAGTTCCGTCTA		822	
	*rplc*-R	GTTGACGCTTTAATGGGCTTA			
*rplD*	*rplD*-F	TCGCTTACCTCCTTAATG		1200	
	*rplD*-R	GGTGGAAACACTGTAACTG			
*rplV*	*rplV*-F	CAACACGAAGTCCGATTGGA’		350	
	*rplV*-R	GCAGACGACAAGAAAACAAG			
*optrA*	*optrA*-F	TACTTGATGAACCTACTAACCA		422	
	*optrA*-R	CCTTGAACTACTGATTCTCGG			
*cfr*	*cfr*-F	TGAAGTATAAAGCAGGTTGGGAGTC		746	
	*cfr*-R	ACCATATAATTGACCACAAGCAGC			
*cfr(B)*	*cfr(B)*-F	TGAGCATATACGAGTAACCTCAAGA’	58	293	
	*cfr(B)*-R	CGCAAGCAGCGTCTATAT CA			
*vanA*	*vanA*-F	ATG AAT AGA ATA AAA GTT GC	50	1032	[43]
	*vanA*-R	TCA CCC CTT TAA CGC TAA TA			
*msrA*	*msrA*-F	GGC ACA ATA AGA GTG TTT AAA GG	40	939	[44]
	*msrA*-R	AAG TTA TAT CAT GAA TAG ATT GTC CTG TT			

#### Sequencing of some selected domains

The PCR products of the genetic fragment of domain V of the 23 S ribosomal RNA, *rplC, rplD* and *rplV* genes were cleaned-up and bidirectionally sequenced by Macrogen Inc., Seoul, Korea via Blutruve, Egypt. The PCR products were extracted using Gel Extraction Kit (PureHelix™ Gel, Korea) and Sanger-sequenced with BigDye terminator v3.1 sequencing kit, and a 3730xl automated sequencer (Applied Biosystems, Foster City, CA, USA). Data obtained from sequencing were aligned and assembled using BioEdit v7.2.5 software to obtain the final consensus. The open reading frames (ORF) of the final contigs for the tested genes were detected by ORF finder (https://www.ncbi.nlm.nih.gov/orffinder/) (accessed 8 August 2022). The sequencing data were analyzed using the basic local alignment search tool BLASTn, BLASTp, uniprot align, and mutation survoyer V5.1.2 (https://blast.ncbi.nlm.nih.gov/Blast.cgi) (accessed 8 August 2022). The relevant sequences of *S. aureus* N315 strain (GenBank, NCBI. Accession no. NC_002745), were used as a reference to detect mutations [46–48].

#### Effect of combining linezolid with some antimicrobials

Six antimicrobial combinations were investigated against LR-MRSA using microtiter plate checkerboard method according to the CLSI M26-A, 1998 protocol [49]. The linezolid was used in combination with each of chloramphenicol, ciprofloxacin, erythromycin, gentamicin, tigecycline, and vancomycin. Firstly, the MICs were determined according to the CLSI guidelines as mentioned previously [28]. A set of serially diluted solutions (1/32 MIC to 4 times MIC) was prepared for every antimicrobial (n = 7) using MHB [50, 51]. A volume of 100 µL aliquots of the first antimicrobial (A) of every dilution was transferred into all the wells of the first column, then a twofold serial dilution was done across the vertical axis of the U-shaped bottom microtiter plate, the same was performed to the second antimicrobial (B) but across the horizontal axis, where each row and column contained constant amount of one agent and decreasing amount of the second one. Finally, an inoculum was transferred to each well to yield a final count of ${10}^{5}$ CFU/mL. A volume of 200 µL MHB was added to well 12H and inoculated to serve as a growth control. The plates were incubated at 37 ℃ for 16–20 h. Eventually, Fractional Inhibitory Concentration Indices (FICIs) were calculated according to the following equation:

$${\text{FICI}}\,{ = }\,{\text{FIC}}_{{\text{A}}} \,{ + }\,{\text{FIC}}_{{\text{B}}} \,{ = }\,{\text{A/MIC}}_{{\text{A}}} \,{ + }\,{\text{B/MIC}}_{{\text{B}}}$$

A and B are the MIC of each antibiotic in combination, and MIC_A_ and MIC_B_ are the MIC of each drug individually. The potencies of combinations were categorized according to FIC indices as: synergism (< 0.5); additive or indifferent effect (0.5–4): antagonism (>4) [22, 51].

### Data and statistical analysis

All experiments were conducted in triplicates except biofilm formation assays which were performed in 8 replicates. Means, medians, and standard error of mean were calculated. All the results were presented as frequencies and percentages. A correlation matrix and correlogram were created to investigate the co-existence of antimicrobial resistance, and Spearman’s correlation coefficients (${\mathrm{r}}_{\mathrm{s}}$) were calculated. Paired Student t-test was used to investigate CCCP effect on linezolid MICs and to compare MICs of linezolid alone and in combination with other antimicrobial. Fisher’s exact (*FE*) test was used to study the association between the phenotype and genotype characters of linezolid resistance. The two-tailed Pearson’s correlation was done for: the linezolid MIC values with the biofilm formation ability and number of de novo mutations; and between FICIs and number of detected mutations. All statistical analysis was done using SPSS for windows v.26.0 (IBM., NY, USA). R-studio version 2022.02.3 for windows (access date: 16/6/2022) was utilized for data visualization using various packages. All data analyses outputs were regarded significant if *p*-value does not exceed 0.05.

## Results

### Isolate collection and characterization

Methicillin-resistant isolates (n = 159) were recovered from various clinical specimens. Isolates were identified as *S. aureus* and cefoxitin disk diffusion assay results have confirmed that all are methicillin-resistant. All isolates were pigment, gelatinase, and catalase producers. Five methicillin-resistant isolates (3.14%, *p* < 0.01) were non-mannitol fermenters on MSA and on *Staphylococcus* medium 110. Thirteen isolates (8.18%, *p* < 0.01) were coagulase negative (MRCoNS).

### Antimicrobial susceptibility testing

#### Antibiogram and MIC

**Fig. 1:**
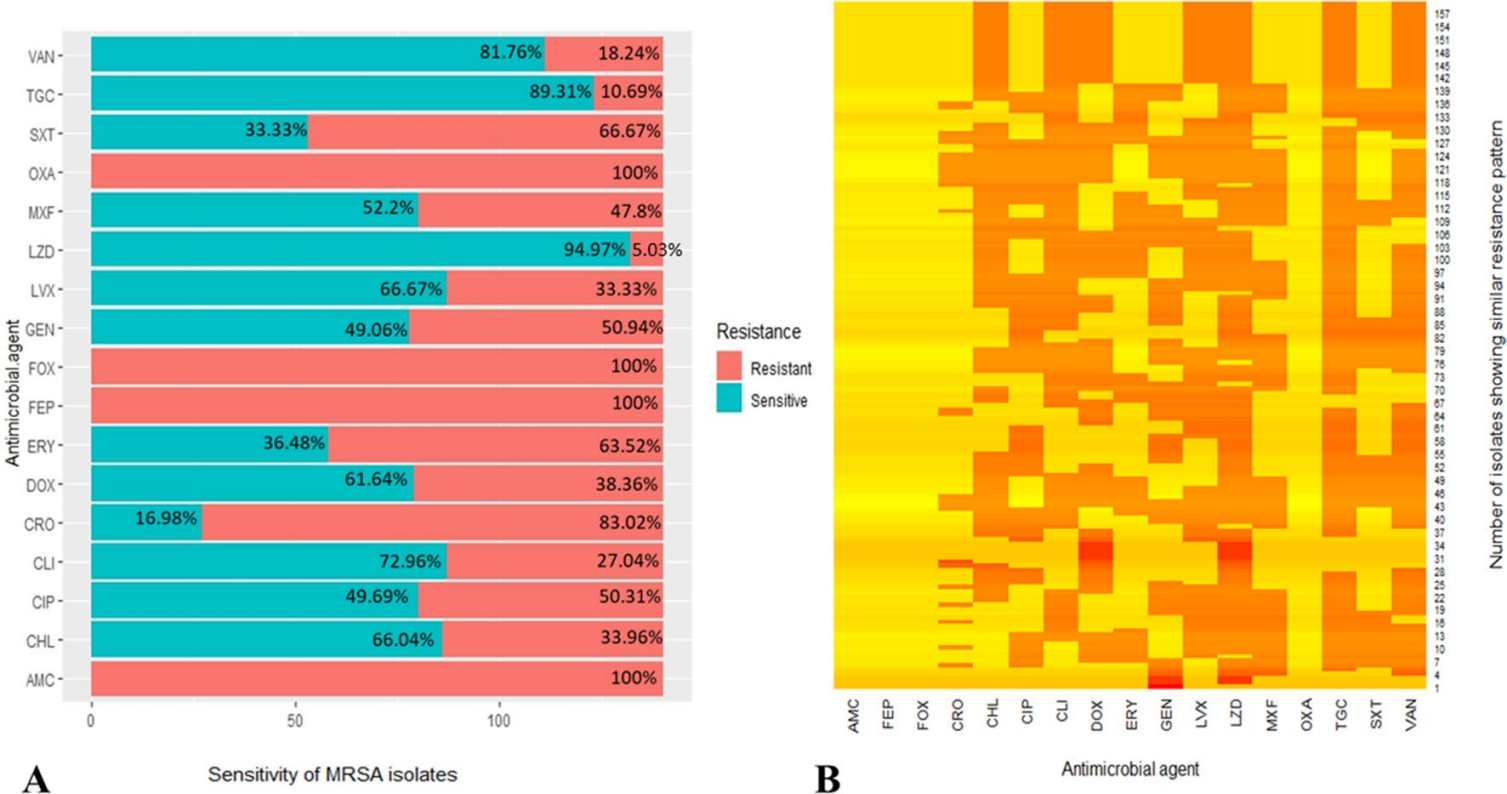
Sensitivity of methicillin-resistant isolates (n = 159) to different antimicrobials. **A** Stacked bar chart representing resistance and sensitivity patterns of different antimicrobials against methicillin-resistant isolates. **B** Heatmap displaying the resistance pattern of each isolate, where yellow color, resistant; orange color, sensitive. *AMC* amoxicillin/clavulanic acid, *CHL* chloramphenicol, *CIP* ciprofloxacin, *CLI* clindamycin, *CRO* ceftriaxone, *DOX* doxycycline, *ERY* erythromycin, *FEP* cefipime, *FOX* Cefoxitin, *GEN* gentamicin, *LVX* levofloxacin, *LZD* linezolid, *MXF* moxifloxacin, *OXA* oxacillin, *SXT* trimethoprim/sulfamethoxazole, *TGC* tigecycline, *VAN* vancomycin. The antimicrobial agents presented as 3 letter abbreviations according to the American Society for Microbiology, (Antimicrobial Agents and Chemotherapy *AAC*)

Among the collected methicillin-resistant isolates (n = 159), 29 isolates (18.24%) were vancomycin-resistant, of which two were MRCoNS. Eight isolates (5.03%) were linezolid resistant (LR-MRSA), of which none were MRCoNS. Linezolid MIC values of the LR-MRSA isolates ranged from 8 to 128 mg/L. These isolates were subject to further phenotypic and genotypic investigation. It is worth mentioning that the 8 LR-MRSA isolates have shown vancomycin resistance, as well. Disk diffusion assay results are shown in Fig. 1. All methicillin-resistant isolates showed resistance to amoxicillin/clavulanic acid, oxacillin, and cefepime. Notably, high levels of resistance were detected to ceftriaxone (83.02%; n = 132), trimethoprim/sulphamethoxazole (66.67%; n = 106), and erythromycin (63.52%; n = 101). Among the collected isolates, 50.94% (n = 81) were resistant to gentamicin, 38.36% (n = 61) were resistant to doxycycline, and 33.96% (n = 54) were chloramphenicol resistant. In addition, relatively high MICs of some other antimicrobials; chloramphenicol, ciprofloxacin, erythromycin, gentamicin, and tigecycline, were observed among the collected isolates (Table 2).

**Table 2:** Distribution of methicillin-resistant isolates (n = 159) based on minimum inhibitory concentrations (MICs) of linezolid, and the other antimicrobials; chloramphenicol, ciprofloxacin, erythromycin, gentamicin, tigecycline, and vancomycin, in mg/L

Antimicrobial	Minimum inhibitory concentration (mg/L)											
	< 2	2	4	8	16	32	64	128	256	512	1024	> 1024
CHL	0	35	25	45	12*	5*	14*	23*	0	0	0	0
CIP	31	48	13*	5*	24*	7*	9*	8*	3*	9*	2*	0
ERY	51	7	21*	43*	3*	12*	18*	4*	0	0	0	0
GEN	0	67	11	5*	2*	37*	5*	9*	11*	5*	7*	0
LZD	0	54	97	2*	1*	1*	1*	3*	0	0	0	0
TGC	142	3*	13*	1*	0	0	0	0	0	0	0	0
VAN	0	17	76	37	3*	2*	10*	8*	3*	3*	0	0

Results were interpreted according to the clinical breakpoints of CLSI, M07-A10 protocol, and to the breakpoint tables version 10.0, 2020 of the European Committee on Antimicrobial Susceptibility Testing (EUCAST) for tigecycline, the resistant isolates are denoted by asterisk

#### Co-occurrence of antimicrobial resistance among the methicillin-resistant isolates

**Fig. 2:**
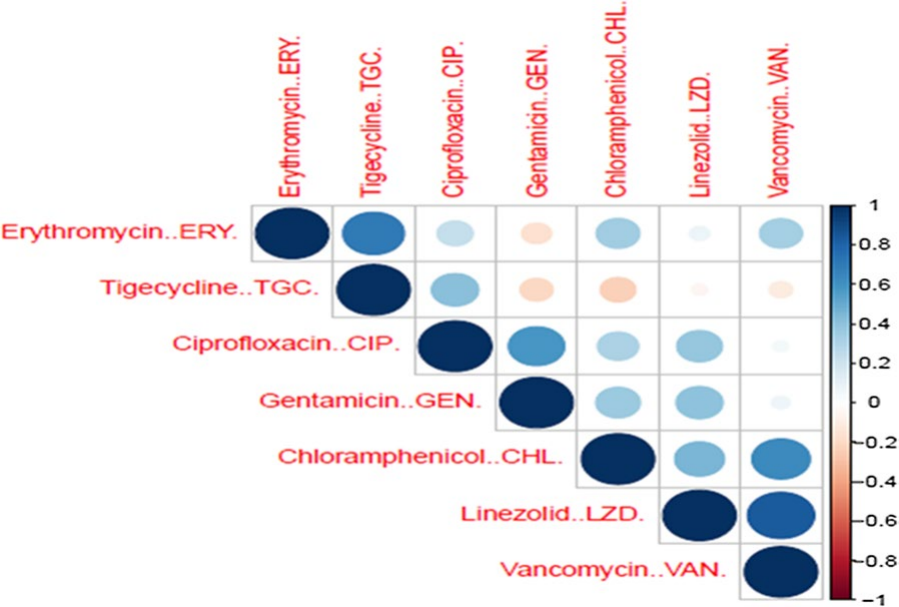
Correlogram representing the antimicrobial-antimicrobial correlations for seven tested antimicrobial agents. Spearman’s correlation coefficients are represented by intensity, where; blue tones indicate positive correlation, and red tones indicate negative correlation

To investigate the co-occurrence of resistance among different antimicrobials, a correlation matrix was constructed based on the MIC values of all methicillin-resistant isolates (n = 159) presented as a correlogram (Fig. 2). Strong positive correlations were observed between resistance to linezolid and vancomycin (${\mathrm{r}}_{s}$ = 0.84, *p* = 0.001), erythromycin and tigycycline (${\mathrm{r}}_{\mathrm{s}}$ = 0.7, *p* = 0.003), chloramphenicol and vancomycin (${\mathrm{r}}_{\mathrm{s}}$ = 0.63, *p* = 0.0097).

### Phenotypic investigation of resistance mechanisms

#### The role of efflux pumps in linezolid resistance

Linezolid MICs were not significantly affected (t = 1.374, *p* = 0.212) by the addition of CCCP. Five LR-MRSA isolates have shown no change in linezolid MIC; 2 isolates showed a twofold decrease; and only one isolate showed a fourfold decrease (Additional file 1: Table S1).

#### Biofilm formation

All the LR-MRSA isolates were biofilm formers. The mean values of the tested isolates were recorded in Table 3. Six isolates showed strong biofilm formation ability and the remaining two were moderate biofilm formers. Strong positive Pearson’s correlation (r = 0.915, *p* = 0.001) was found between linezolid MICs and mean of the biofilm formation.

**Table 3:** Biofilm formation quantification of LR-MRSA isolates by crystal violet staining microtiter plate method, Mean optical density (OD) ± standard error of mean (SEM), in correlation with their linezolid MIC values

Isolate Code	Mean optical density (OD) ± SEM	Categorization	Linezolid MICs (mg/L)
9A	7.69 ± 0.108	Strong biofilm producer	128
57A	4.95 ± 0.024	Strong biofilm producer	32
90A	6.98 ± 0.093	Strong biofilm producer	128
95A	3.8 ± 0.074	Strong biofilm producer	8
112A	6.94 ± 0.312	Strong biofilm producer	64
117A	7.32 ± 0.421	Strong biofilm producer	128
126A	2.5 ± 0.073	Moderate biofilm producer	8
137A	2.81 ± 0.053	Moderate biofilm producer	16

### Genotypic characterization

#### Molecular detection of some resistance genes

**Fig. 3:**
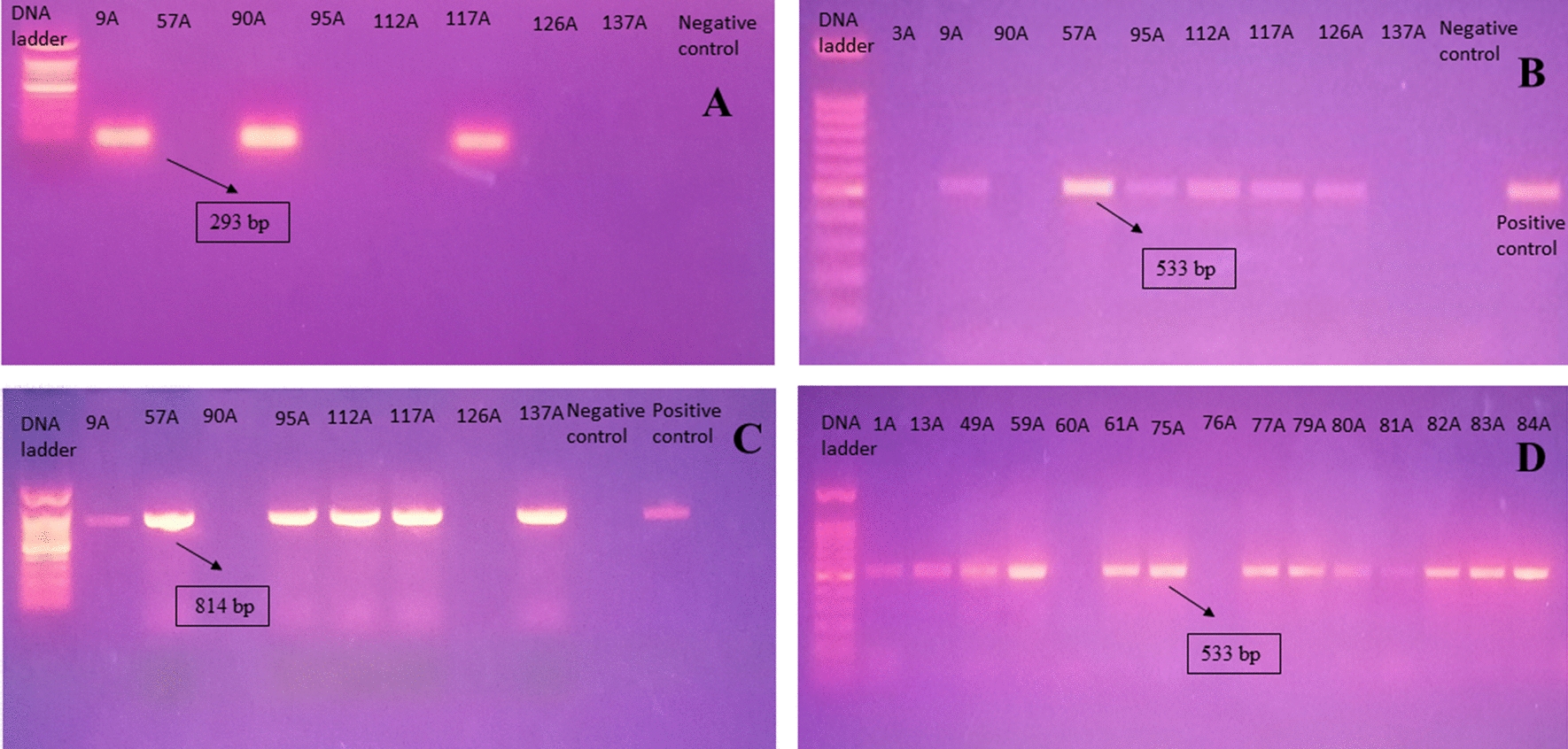
Agarose gel electrophoresis of some PCR products detected among LR-MRSA isolates (n = 8). **A** *cfr*(B) gene (293 bp); nuclease free water as a negative control. **B** *mecA* gene (533 bp) and *S. aureus* (MSSA) ATCC^®^ 25923 and; *S. aureus* (MRSA) ATCC^®^ 43300 as negative and positive controls, respectively. **C** *icaA* gene (814 bp); and nuclease free water as a negative control and *S. aureus* ATCC^®^ 43300 as a positive control. **D** *mecA* gene (533 bp) in VRSA isolates

Domain V of the 23 S rRNA, *rplC*, *rplD*, *rplV*, and *vanA* genes were detected in all the LR-MRSA isolates. On the other hand, *optrA*, *cfr,* and *msrA* genes were not detected in any of the isolates. Three isolates harbored *cfr(B)* gene (Fig. 3A), both *mecA* (Fig. 3B) and *icaA* genes (Fig. 3C) were detected in six LR-MRSA isolates. Nonetheless, no plasmids were detected in any of LR-MRSA isolates. It is worth mentioning that when looking at all methicillin-resistant isolates (n = 159), *mecA* gene was detected in 92.45% (n = 147) of the isolates. Among the VRSA isolates (n = 29), 93.10% (n = 27) isolates harbored *mecA* (Fig. 3D) and 37.93% (n = 11) harbored *vanA* genes, respectively.

#### Sequencing

Some mutations in domain V region of 23 S rRNA were detected in LR-MRSA isolates, A2338T and C2610G mutations were observed in 5 LR-MRSA isolates; two isolates showed T2504C and G2528C mutations; and only one isolate harbored G2576T mutation. Out of the 8 LR-MRSA isolates, 3 harbored mutations in amino acid coding regions of L3 (*rplC* gene). L4 (*rplD* gene) mutations were detected in half of isolates (n = 4). Furthermore, no L22 (*rplV* gene) mutations were detected in any isolate. Detected resistance-related genes or mutations in LR-MRSA isolates (n = 8) with their linezolid MICs are illustrated in Table 4.

**Table 4:** Genotypic characterization of linezolid resistance mechanism(s) in LR-MRSA isolates (n = 8) as revealed by numerous point mutations in the target location

Isolate Code	Linezolid resistance gene(s)	Linezolid (MICs; mg/L)
9A	*cfr(B), vanA, mecA, icaA,* domain V region of 23S rRNA mutations* (*A2338T and C2610G)	128
57A	*vanA, mecA, icaA,* L3 *(rplC* gene) mutations (Ser124Leu, Ile215Asn), and domain V region of 23S rRNA mutations* (*A2338T, and C2610G)	32
90A	*cfr(B), vanA,* L3 *(rplC* gene) mutations (Gly75Thr, Thr179Ala, Ile215Asn), and domain V region of 23S rRNA mutations (A2338T, C2610G, G2482T, C2493A, A2498C, T2504C, G2528C, T2531C, C2534A, C2548G, T2555C, G2576T, G2603T, G2604T, T2607C)	128
95A	*vanA, mecA, icaA,* L3 *(rplC* gene) mutations, (Ile215Asn), and domain V region of 23S rRNA mutations (C2610G, T2504C, G2528C, G2589T)	8
112A	*vanA, mecA, icaA, L4 (rplD* gene) mutations (Leu2Tyr, Phe3Ser, Glu4Lys), and domain V region of 23S rRNA mutations (A2338T)	64
117A	*cfr(B), vanA, mecA, icaA, and L4 (rplD* gene) mutations (Pro29Gln, Asn30Ile, Leu34Tyr)	128
126A	*vanA, mecA, icaA,* and L4* (rplD* gene) mutations (Glu28Ser)	8
137A	*vanA,* L4* (rplD* gene) mutations (Pro29Gln, Ser32Ala, Leu34Tyr), and domain V region of 23S rRNA mutations (A2338T, C2610G)	16

#### Genotypic-phenotypic correlation of linezolid resistance mechanisms among LR-MRSA isolates

No significant correlations (*p* > 0.05) were observed between linezolid MIC values and number of mutations detected in domain V region of 23 S rRNA or L3 (*rplc* gene), with Pearson’s correlation coefficients: r = 0.45 and r = 0.281, respectively (Additional file 2: Table S2). Despite high linezolid MICs median value (median = 128, *FE p* = 0.089) of isolates harboring *cfr(B)* gene, no significant correlation was detected between linezolid MICs and presence of *cfr(B)* gene. Intriguingly, no statistical correlation was detected between linezolid resistance molecular mechanisms and the biofilm forming ability among the LR-MRSA isolates (*FE p* > 0.05). Detected mutations and resistance-related genes in relation to biofilm production with their Fisher’s exact *p*-values are listed in Table 5.

**Table 5:** Association between the genotypic resistance mechanisms and phenotypic biofilm formation ability of the 8 LR-MRSA isolates

Genotype	No. of isolates	Biofilm formation ability			Fisher’s exact test
		Strong	Moderate	Weak	
Domain V region of 23S rRNA mutations	6	5	1	–	P = 0.464
*rplC* gene (L3) mutations	3	3	–	–	P = 0.357
*rplD* gene (L4) mutations	4	2	2	–	P = 0.214
*rplV* gene (L22) mutations	–	–	–	–	–
*cfr*	–	–	–	–	–
*cfr(B)*	3	3	–	–	P = 0.357
*optrA*	–	–	–	–	–
*msrA*	–	–	–	–	–
*icaA*	6	5	1	–	P = 0.464
*vanA*	8	6	2	–	–
*mecA*	6	6	2	–	P = 0.464

#### Combining linezolid with other antimicrobial agents to combat LR-MRSA isolates

**Fig. 4:**
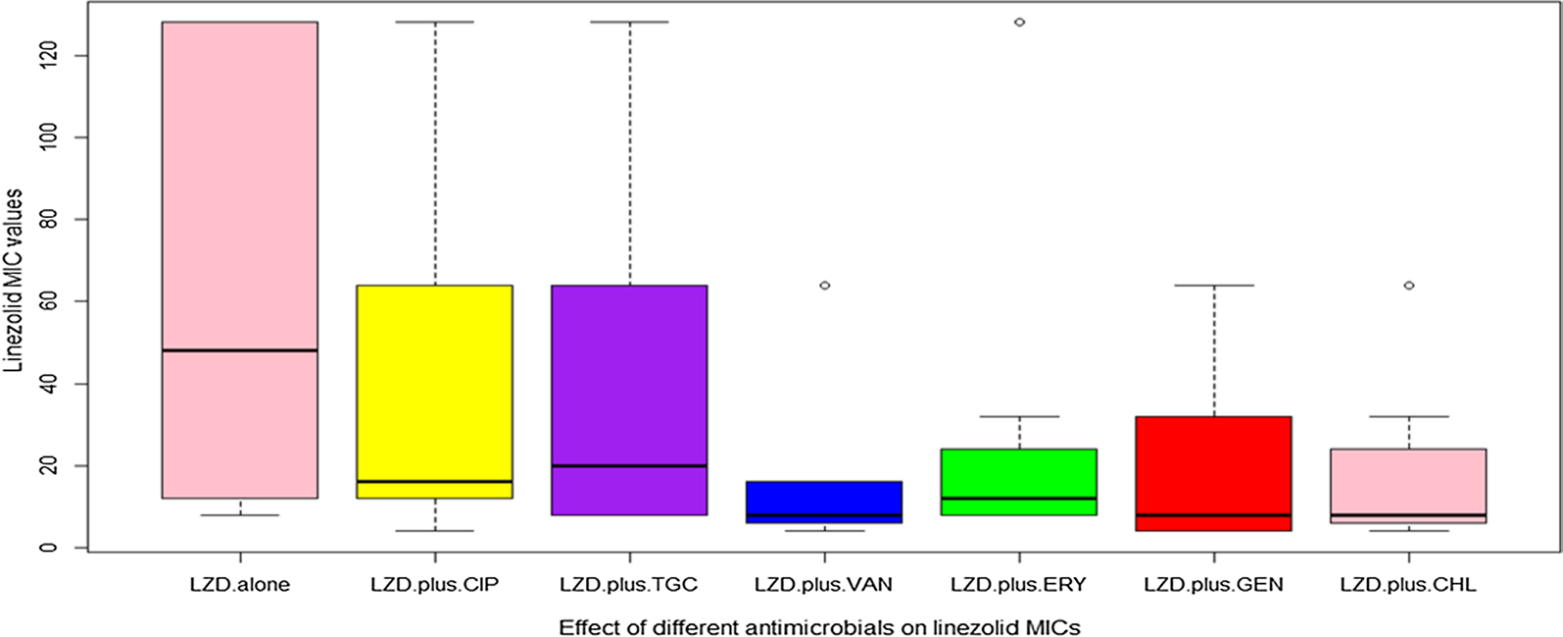
Graphical representation of linezolid MIC values against LR-MRSA isolates (n = 8) compared to its values when combined with other antimicrobials, median values were calculated; LZD MICs median = 48, LZD MICs in presence of CIP median = 16, LZD MICs in presence of TGC median = 20, LZD MICs in presence of VAN median = 8, LZD MICs in presence of ERY median = 12, LZD MICs in presence of GEN median = 8, and LZD MICs in presence of CHL median = 8

**Fig. 5:**
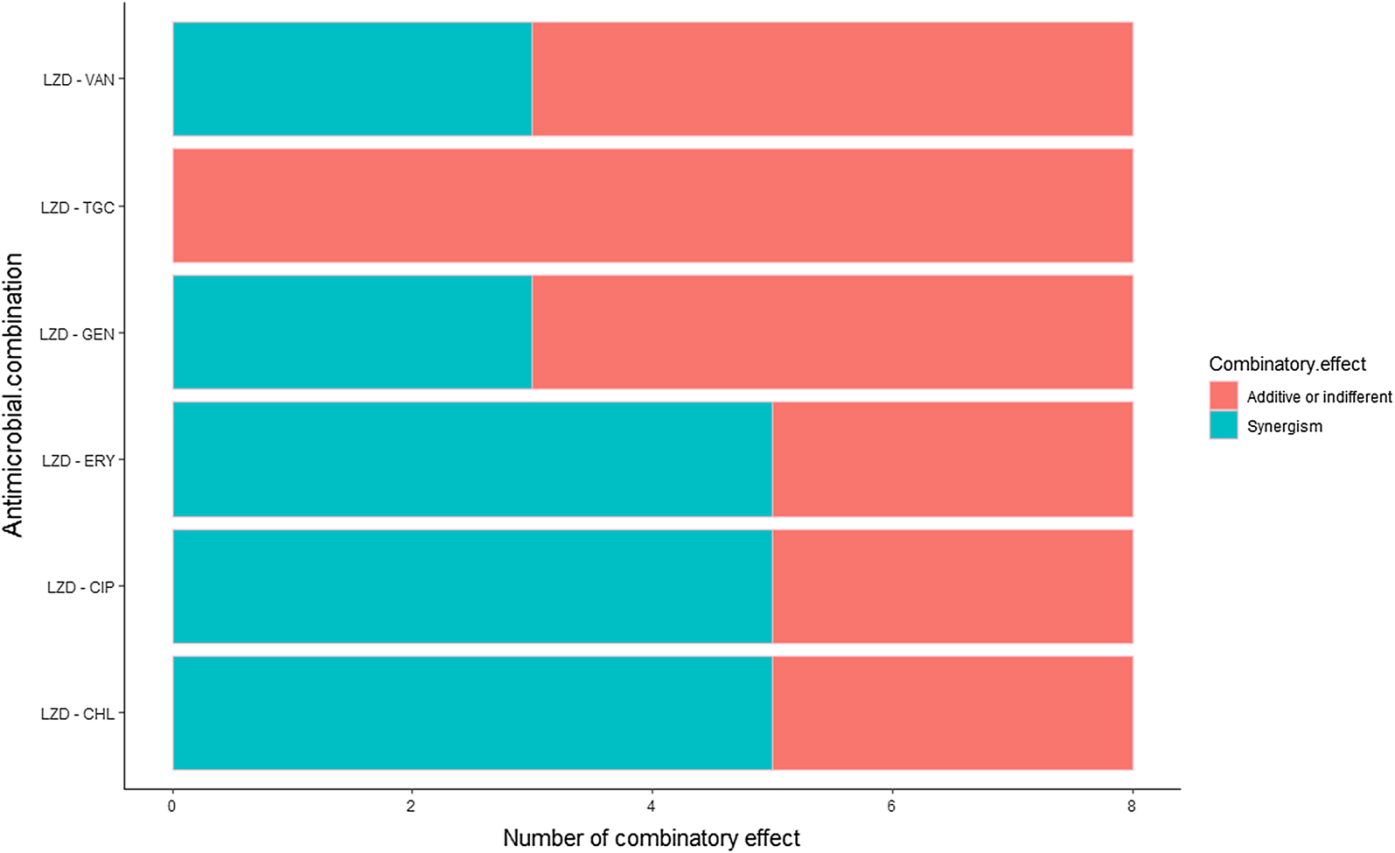
Effect of different antimicrobial combinations on LR-MRSA isolates (n = 8)

Linezolid MICs of LR-MRSA were studied in combination with some antimicrobials and the median values were calculated (Fig. 4). Of the 6 antimicrobial combinations tested, three combinations showed synergism against five isolates (FICIs < 0.5); linezolid-chloramphenicol (t = 3.05**,** *p* = 0.019), linezolid-erythromycin (t = 2.105, *p* = 0.037), and linezolid-ciprofloxacin (t = 2.349, *p* = 0.05). In addition, linezolid-gentamicin combination reversed linezolid resistance in 2 isolates (t = 3.238, *p* = 0.014), and linezolid-vancomycin combination reversed linezolid resistance in 3 isolates (t = 2.809, *p* = 0.026). Effect of different antimicrobial combinations on LR-MRSA isolates is presented as a stacked bar chart in Fig. 5. MICs of the used antimicrobial agents, FICIs, and their interpretations are listed in Additional file 3: Table S3 and Additional file 4: Table S4.

#### Association between synergistic effect of antimicrobial combinations and genetic characteristics

Synergistic effect of combinations in association with detected molecular mechanisms among LR-MRSA isolates are represented in Table 6. A statistically significant association was observed between synergistic effect of linezolid-gentamicin (LZD-GEN) combination and presence of *cfr(B)* gene (*FE* test, *p* = 0.008), 2 isolates harboring *cfr(B)* have shown reduction in their MIC values when subjected to LZD—GEN combination (FICIs were 0.39, and 0.25). In addition, positive correlation was observed between FICIs of this combination and number of mutations in L4 (*rplD* gene, r = 0.239). Positive correlation was recorded between linezolid-ciprofloxacin (LZD—CIP) combination FICI values and number of mutations in L4 (*rplD* gene, r = 0.402). Linezolid-vancomycin (LZD—VAN) combination FICIs positively correlated with number of mutations detected in L3 (*rplC* gene, r = 0.409).

**Table 6:** Tabular representation of number of isolates showing synergism in association with the molecular resistance mechanisms among LR-MRSA isolates

Synergistic combinations	Number of LR-MRSA isolates showing synergism	Number of isolates showing resistance genotype					
		Domain V region of 23 S rRNA mutations	*rplC* gene (L3) mutations	*rplD* gene (L4) mutations	*cfr(B)*	*icaA*	*mecA*
LZD—CHL	5	4	3	2	2	3	3
LZD—GEN	3	2	2	1	3	2	2
LZD—ERY	5	4	3	2	3	4	4
LZD—VAN	3	3	2	1	1	3	3
LZD—CIP	5	4	3	2	1	3	3

## Discussion

For years, there has been an increase in the global medical interest in finding new options to manage MRSA infections. Recently, linezolid has been prescribed intensively for some severe infections as hospital and community-acquired pneumonia (HAP and CAP), acute MRSA infections as well as acute skin and skin-structure infections (SSSIs) [5]. However, reduced susceptibility to linezolid has started to appear gradually in clinical Gram-positive isolates [52–55]. Accordingly, this study was an attempt to elucidate various mechanisms of linezolid resistance adopted by MRSA clinical isolates.

Several studies proved that linezolid had superior efficacy in managing MRSA infections and may be the drug of choice [6, 9, 13, 56]. Our results showed that only 5.48% of the isolates were insensitive to linezolid (LR-MRSA). Yet still limited, this percentage exceeds other recently reported studies. In 2020, Maarouf et al*.* [46] studied 232 *Staphylococcal* isolates retrieved from Alexandria Main hospital and reported that 1.3% possessed elevated linezolid MICs (128–256 µg/mL) [46]. Also, prevalence of linezolid resistance was 2.74% among Gram-positive isolates in Tanta University Hospital, as reported by Abdelkhalek et al*.* 2021 [57]. In the same context, linezolid resistance of Staphylococci isolates obtained from 15 different countries were 0.1% and 0.3% among MRSA and CoNS isolates, respectively [7]. However, some studies conducted in other countries reported complete susceptibility of MRSA to linezolid [58–65].

Twenty-seven MRSA isolates were resistant to vancomycin. The first MRSA showing vancomycin resistance was detected in 1996 in Japan after prolonged treatment [55]. Similarly, Saeed et al*.* 2019 [66] reported the co-resistance of methicillin and vancomycin in 14 of 100 clinically collected *S. aureus* isolates [66]. According to Shariati et al. [67] the overall global prevalence of vancomycin resistance in the previous 20 years was 1.5% among *S. aureus* isolates and the highest prevalence 3.6% was detected in U.S [67]. Diverse vancomycin resistance strategies are developed by Gram-positive bacteria, mainly cell wall alterations encoded by *vanA* cluster integrated within Tn1546 [68]. Staphylococci may acquire vancomycin reduced susceptibility by Tn1546 *Enterococcal* transposon transfer [68].

Among the tested isolates, statistically significant correlations were detected between vancomycin resistance and each of linezolid and chloramphenicol resistance. Similarly, Yadav et al*.* 2017 [69] observed co-resistance to linezolid and vancomycin in 2% of 200 *Enterococcus* strains, in addition to high level of gentamicin resistance [69]. Abbo et al*.* 2019 [70] isolated 4 LR-VRE isolates from patients admitted to the intensive care unit (ICU) of tertiary care teaching hospital in Miami, Florida [70]. Although linezolid and vancomycin exert their actions by completely different mechanisms, co-resistance to them may be a consequence of *vanA* gene expression in the tested isolates. *vanA* gene is responsible for replacement of D-alanine (D-Ala) terminal with D-lactate (D-Lac) during cell wall formation which imparts vancomycin insensitivity in Gram-positive isolates [71]. Our results showed significant correlations between erythromycin and tigycycline resistance among methicillin-resistant isolates. Resistance to both macrolides and tetracyclines may be mediated through efflux pumps activity encoded mainly by *mef(A), tet(K)*, or *tet(L)* genes, which usually altogether are harbored by the same mobile genetic elements [72, 73].

LR-MRSA isolates were investigated for some phenotypic factors which may mediate or amplify linezolid resistance in Gram-positive isolates. Upregulation of efflux pumps is one of the known mechanisms responsible for resistance in bacteria as it prevents accumulation of antibacterial agents inside the cell and may amplify isolate resistance if it combines with other mechanisms [32]. About fifteen different efflux pumps were detected in *S. aureus* isolates [74].Various efflux pumps and transporters have been reported to induce reduced susceptibility to linezolid among Gram-positive isolates as: (i) ABC transporters encoded by *optrA* gene which is usually co-found with *cfr* gene or its homologues *cfr(B)* and *cfr(C)* genes [21]. Moreover, ABC transporter optrA was detected in 6.55% of 885 *Enterococcus* spp. isolates with increased oxazolidinone and chloramphenicol MICs [75]. (ii) MsrA protein which is considered as one of ATP transporter family coded by *msrA* gene which may amplify virulence and induce resistance among Staphylococci [16], and (iii) LmrS efflux pump, a member of major facilitator superfamily (MFS), which actively extrudes different antimicrobials of various structures and mechanisms of action including linezolid [76]. The role of efflux pump upregulation was tested using CCCP, the commonly used non-specific efflux pump inhibitor that alters the proton gradient against the cell membranes [32]. Significant participation of efflux pumps in resistance is denoted by at least an eightfold decrease in MIC in presence of CCCP [32, 33]. None of the LR-MRSA isolates enrolled in this study showed a significant activity for efflux pumps that can be inhibited by CCCP. Usually, efflux pumps show more contribution to linezolid resistance among Gram-negative rather than Gram-positive bacteria [77]. Higher levels of linezolid accumulated in *S. aureus* and *E. faecium* strains than in *E. coli, Citrobacter freundii* and *Enterobacter aerogenes* strains. Linezolid accumulation levels in Gram-negative strains increased in presence of CCCP, while were not significantly affected in Gram-positive strains [77].

Biofilms are a primary concern while handling any chronic or recurrent microbial infection especially implant/device related ones [38]. Biofilm aggregates are very difficult to penetrate being composed mainly of slime like substances as polysaccharide intercellular adhesions (PIA) encoded by *icaA* operon in *S. aureus* [37, 38]. All LR-MRSA isolates showed biofilm formation ability and *icaA* gene was detected in six isolates of them, *icaA* gene is responsible for production of N-acetylglucosamyl transferase enzyme, which contributes to PIA formation [45]. A statistical significant correlation was observed between linezolid MIC values and the mean biofilm formation. Similarly, Zheng et al*.* 2017 [17] found higher biofilm formation in linezolid resistant *E. faecalis* clinical isolates than the sensitive ones [17]. Besides, some other studies showed significant correlation between biofilm formation and induction of multi-drug resistance among Gram-positive isolates [4, 78].

Prevalence of *mecA* gene among methicillin-resistant isolates was investigated showing that 92.45% of MRSA isolates including six LR-MRSA isolates harbored* mecA* gene that codes for low-affinity penicillin binding protein (PBP2a). Similarly, Elhassan et al*.* 2015 [79] and Hawraa et al*.* 2014 [80] reported that 9.8% and 28.95% of phenotypically confirmed MRSA were* mecA* gene negative which may suggest presence of other resistance mechanisms as hyper-production of β- lactamase [79, 80]. In the same context, Ba et al*.* 2014 [81] stated that PBPs 1, 2, and 3 amino acid alterations mediate resistance in phenotypic MRSA-*mecA* negative clinical isolates [81]. Vancomycin resistance gene *vanA* was detected in 6.92% (n = 11) of methicillin-resistant isolates (n = 159) and eight of them showed linezolid insensitivity, as well. Among 30 MRSA isolates with concurrent vancomycin resistance or intermediate resistance (VRSA/VISA), *vanA* gene was detected in 14 isolates of them [66].

Genotypic characterization was done through detection of some linezolid related resistance genes (*cfr*, *cfr(B)*, *msr A*, and *optr A*). In this study, *cfr(B)* gene was the only detected gene in LR-MRSA isolates (n = 3). *cfr(B)* gene mediated the same resistance profile of *cfr* gene when integrated and expressed in *S. aureus* [19]. *cfr* gene methylates A2503 site in 23 S rRNA gene copies imparting steric hindrance in the linezolid fitting site [14, 19], it was first detected in a LR-MRSA infection in 2005 [10, 82]. To the best of our knowledge, this is the first report on the detection of* cfr(B)* gene in clinically isolated MRSA in Egypt. The only amplified linezolid resistance gene in Egyptian hospitals was *cfr* gene [46, 57].

Linezolid binds to PTC of 23 S rRNA gene to confer antibacterial effect, its resistance can be mediated via development of mutations in domain V of 23 S rRNA or ribosomal proteins located near the linezolid binding site in PTC (L3, L4, or L22) [6]. The 23 S rRNA domain V and ribosomal protein coding genes (*rplC*, *rplD*, and *rplV*) were detected in all LR-MRSA isolates, their PCR products were extracted and Sanger sequenced. In this study, domain V of 23 S rRNA mutations were observed in 5 LR-MRSA isolates, A2338T and C2610G showed the highest prevalence. Furthermore, 2 LR-MRSA isolates have T2504C and G2528C mutations, and G2576T mutation detected in one isolate. G2576T is the most frequently reported mutation in domain V of 23 S rRNA among linezolid resistant MRSA isolates after prolonged exposure [6]. Other mutations as; T2500A, G2447T, and G2592T in 23 S rRNA genes were reported by some other studies in different linezolid resistant Gram-positive isolates [6, 9, 10, 20, 56]. In accordance to our results, Maarouf et al*.* 2021 [46] has found G2603T mutation in domain V in 2 linezolid resistant isolates out of 232 clinical *Staphylococcal* isolates obtained from Alexandria Main University Hospital, Egypt [46]. Ribosomal proteins of large ribosome subunit as L3, L4, and L22 proteins located near the PTC usually contribute to core structure stability of ribosomes and interact with various domains and RNA elements [83]. L3 and L4 proteins contribute in PTC formation [83]. L22 protein has the ability to interact with the six different domains of 23 S rRNA and affects the conformation and folding of 23 S rRNA [84]. L3 amino acid substitution Ile215Asn, and L4 amino acid substitutions Pro29Gln and Leu34Tyr have shown the highest prevalence among LR-MRSA isolates. In agreement with Yoo et al*.* 2019 [9], no mutations have been detected in L22 protein of LR-MRSA isolates [9].

One of the aims of this study is to investigate the correlation between genotypic and phenotypic mechanisms of linezolid resistance among LR-MRSA isolates. Notably increased MIC values were found in isolates harboring *cfr(B)* gene. This may be due to the presence of some mutations in the genes for ribosomal proteins in these isolates [8].

Adopting antimicrobial combination therapy in clinical practice is a promising strategy to overcome polymicrobial and multi-drug resistant infections [22]. In this study, linezolid was tested in combination with 6 antimicrobial agents. Auspiciously, combinations of linezolid with chloramphenicol, erythromycin, and ciprofloxacin showed synergistic activity against 5 LR-MRSA isolates. Linezolid and erythromycin co-administration was synergistic against Gram-positive isolates, while linezolid and chloramphenicol showed indifferent effect [25]. In this study, Linezolid and chloramphenicol combination showed synergism in 2 LR-MRSA isolates harboring *cfr(B)* gene, which may suggest that *cfr(B)* gene unlike to *cfr* gene is not related to PhLOPSA resistance phenotype [9]. Ciprofloxacin differs greatly in mechanism of action from linezolid, it is a member of fluoroquinolones acting on DNA topoisomerase preventing DNA replication [85]. Indifferent effect was detected upon testing the combination ciprofloxacin and linezolid in *S. aureus* [85, 86].

Linezolid combination with vancomycin showed synergistic effect against 3 LR-MRSA isolates. This combination might be an option for LR-MRSA infections treatment, where their FICIs correlated with number of mutations of L3 ribosomal protein. This may be a consequence of vancomycin ability to inhibit cell wall formation [67]. Also, co-administration of linezolid and cell wall inhibitors, fosfomycin or imipenem showed synergistic activity in 50% and 90% of linezolid resistant MRSA isolates [22]. Linezolid-gentamicin combination showed indifferent effect in 5 LR-MRSA isolates, while this combination was synergistic against 3 LR-MRSA isolates. Previous studies reported indifferent antibacterial action of this combination in *S. aureus* isolates resistant or sensitive to methicillin [22, 24, 87]. Luckily, this combination showed significant synergism in 2 LR-MRSA isolates harboring linezolid resistance gene *cfr(B)*. The FICI values of this combination positively correlated with number of mutations detected in L4 protein. Apparently, gentamicin addition to linezolid can overcome LR-MRSA infections as gentamicin is an aminoglycoside member, which has different binding site than linezolid, where gentamicin exerts its action via binding to the aminoacyl center of 16 S RNA of 30 S ribosomal subunit leading to protein synthesis inhibition [22].

The synergistic interaction of different antimicrobial agents exact mechanisms are not fully understood and may vary with the strain type [87]. Further studies both in vitro and in vivo are needed to be conducted to assess the different possibilities of antimicrobial combinations to be used against multi-drug-resistant infections.

## Conclusion

From this study, it can be concluded that, MRSA isolates show increasing multidrug-resistance pattern including resistance to synthetic antimicrobials as linezolid. Linezolid resistance was detected in 5.48% of the collected MRSA isolates. This study highlighted the significant association between linezolid MICs and biofilm formation. Besides, positive correlations were recorded between linezolid MIC values and number of mutations in both domain V of 23 S rRNA and *rplc* gene, coding for L3 protein. The results illustrated the significant impact of *cfr(B)* gene on linezolid resistance among LR-MRSA isolates. In addition, this study illustrated that linezolid combinations with some antibiotics (gentamicin, vancomycin, chloramphenicol, erythromycin or ciprofloxacin) showed synergistic effect against LR-MRSA isolates.

## Supplementary Information

**Additional file 1: Table S1.** The minimum inhibitory concentrations (MICs), mg/L of LR-MRSA isolates (n = 8) in absence, and presence of CCCP and their MIC fold reduction.

**Additional file 2: Table S2.** Number of mutations in association with the corresponding linezolid MICs of 8 LR-MRSA isolates.

**Additional file 3: Table S3.** Minimum inhibitory concentrations (MICs) of linezolid and other antimicrobials, in mg/L against LR-MRSA isolates (n=8).

**Additional file 4: Table S4.** Checkerboard assay results for the combinations of linezolid with other antimicrobials against LR-MRSA isolates (n=8).

## Data Availability

All data generated or analysed during this study are included in this published article and its supplementary information files.

## Abbreviations

AAC: Antimicrobial Agents and Chemotherapy
ABC: ATP-binding cassette transporter protein
AMC: Amoxicillin/clavulanic acid
API: Analytical profile index
ATCC: American Type Culture Collection
BMD: Broth micro-dilution method
BLAST: Basic Local Alignment Search Tool
bp: Base pair
CAMHB: Cation adjusted Mueller Hinton broth
CAP: Community-acquired pneumonia
CCCP: Carbonyl cyanide m-chlorophenylhydrazone
CFU: Colony forming unit
CHL: Chloramphenicol
CIP: Ciprofloxacin
CLI: Clindamycin
CLSI: Clinical and Laboratory Standards Institute
CoNS: Coagulase-negative Staphylococci
CRO: Ceftriaxone
CV: Crystal violet
D-ala: D-alanin
DNA: Deoxyribonucleic acid
D-lac: D-lactate
DOX: Doxycycline
ERY: Erythromycin
EUCAST: European Committee on Antimicrobial Susceptibility Testing
FDA: Food and drug administration
FE: Fisher’s exact
FEP: Cefipime
FIC: Fractional inhibitory concentration
FICI: Fractional inhibitory concentration index
FOX: Cefoxitin
GEN: Gentamicin
HAP: Hospital-acquired pneumonia
ICU: Intensive care unit
IZ: Inhibition zone
Kb: Kilo base pair
LR-CoNS: Linezolid-resistant coagulase-negative Staphylococci
LR-MRSA: Linezolid-resistant MRSA
LRSA: Linezolid-resistant *S. aureus*
LR-VRE: Linezolid-resistant vancomycin-resistant Enterococci
LVX: Levofloxacin
LZD: Linezolid
MFS: Major facilitator superfamily
MIC: Minimum inhibitory concentration
MRCoNS: Methicillin-resistant coagulase negative Staphylococci
MRSA: Methicillin-resistant *Staphylococcus aureus*
MSA: Mannitol salt agar
MsrA: Macrolides related resistance ABC transporter
MSSA: Methicillin sensitive *S. aureus*
MXF: Moxifloxacin
OD: Optical density
ODc: Optical density of negative control
ORF: Open reading frame
OXA: Oxacillin
PBP2a: Penicillin binding protein 2a
PBS: Phosphate buffered saline
PCR: Polymerase chain reaction
PhLOPSA: (Resistance phenotype of: phenicol; lincosamide; oxazolidinone; pleuromutilin; and streptograminA)
PIA: Polysaccharide intercellular adhesions
PTC: Peptidyl transferase center
rRNA: Ribosomal ribonucleic acid
RTU: Ready to use
SEM: Standard error of mean
SSSIs: Skin and skin structure infections
Ta: Annealing temperature
TGC: Tigecycline
tRNA: Transfer ribonucleic acid
TSB: Tryptic Soy broth
SXT: Trimethoprim/sulfamethoxazole
US: United states
VAN: Vancomycin
VAP: Ventillator-associated pneumonia
VISA: Vancomycin-intermedium resistant
VRE: Vancomycin-resistant Enterococci
VRSA: Vancomycin-resistant *Staphylococcus aureus*
