# Supplementary material for: Phenotypic and genotypic characterization of linezolid resistance and the effect of antibiotic combinations on methicillin-resistant Staphylococcus aureus clinical isolates
Source: Ann Clin Microbiol Antimicrob. 2023 Apr 3;22:23. doi: 10.1186/s12941-023-00574-2 (PMC10069030; doi:10.1186/s12941-023-00574-2)
Supplement: Supplementary file 1 — Additional file 1: Table S1. The minimum inhibitory concentrations (MICs), mg/L of LR-MRSA isolates (n = 8) in absence, and presence of CCCP and their MIC fold reduction. [file 12941_2023_574_MOESM1_ESM.docx]

**Table S1.** The minimum inhibitory concentrations (MICs), mg/L of LR-MRSA isolates (n = 8) in absence, presence of CCCP, and their MIC fold reduction.

| **Isolate code** | **Linezolid Minimum inhibitory concentration (MICs), mg/L** | **CCCP Minimum inhibitory concentration (MICs), mg/L** | **Linezolid Minimum inhibitory concentration (MICs), mg/L in presence of CCCP** | **MIC fold reduction** |
| --- | --- | --- | --- | --- |
| 9A | 128 | 4 | 128 | 1 |
| 57A | 32 | 2 | 32 | 1 |
| 90A | 128 | 8 | 32 | 4 |
| 95A | 8 | 2 | 8 | 1 |
| 112A | 64 | 2 | 32 | 2 |
| 117A | 128 | 1 | 128 | 1 |
| 126A | 8 | 1 | 4 | 2 |
| 137A | 16 | 2 | 16 | 1 |
