# Supplementary material for: Phenotypic and genotypic characterization of linezolid resistance and the effect of antibiotic combinations on methicillin-resistant Staphylococcus aureus clinical isolates
Source: Ann Clin Microbiol Antimicrob. 2023 Apr 3;22:23. doi: 10.1186/s12941-023-00574-2 (PMC10069030; doi:10.1186/s12941-023-00574-2)
Supplement: Supplementary file 2 — Additional file 2: Table S2. Number of mutations in association with the corresponding linezolid MICs of 8 LR-MRSA isolates. [file 12941_2023_574_MOESM2_ESM.docx]

**Table S2.** Number of mutations in association with the corresponding linezolid MICs of 8 LR-MRSA isolates.

| **Resistance genotype** | **No. of isolates** | **Linezolid minimum inhibitory concentrations (MICs), mg/L** | | | | | **Pearson’s correlation between linezolid MIC and resistance genotype** |
| --- | --- | --- | --- | --- | --- | --- | --- |
|  |  | **8** | **16** | **32** | **64** | **128** |  |
| Domain V region of 23S rRNA mutations gene | 6 | 1 | 1 | 1 | 1 | 2 | r = 0.45, *p* = 0.26 |
| *L3*(*rplC* gene) mutations | 3 | 1 | - | 1 | - | 1 | r = 0.281, *p* = 0.5 |
| L4 (*rplD* gene) mutations | 4 | 1 | 1 | - | 1 | 1 | r = -0.302, *p* = 0.466 |
| L22 (*rplV* gene) mutations | - | - | - | - | - | - | - |
