# Supplementary material for: Phenotypic and genotypic characterization of linezolid resistance and the effect of antibiotic combinations on methicillin-resistant Staphylococcus aureus clinical isolates
Source: Ann Clin Microbiol Antimicrob. 2023 Apr 3;22:23. doi: 10.1186/s12941-023-00574-2 (PMC10069030; doi:10.1186/s12941-023-00574-2)
Supplement: Supplementary file 3 — Additional file 3: Table S3. Minimum inhibitory concentrations (MICs) of linezolid and other antimicrobials, in mg/L against LR-MRSA isolates (n=8). [file 12941_2023_574_MOESM3_ESM.docx]

**Table S3.** Minimum inhibitory concentrations (MICs) of linezolid and other antimicrobials, in mg/L against LR-MRSA isolates (n=8).

| **Isolate Code** | **Minimum inhibitory concentrations (MICs), mg/L (Breakpoint)** | | | | | | | |
| --- | --- | --- | --- | --- | --- | --- | --- | --- |
|  | **CRO (8)** | **CHL (32)** | **CIP (4)** | **ERY (8)** | **GEN (16)** | **LZD (8)** | **TGC (0.5)** | **VAN (16)** |
| 9A | 256* | 128* | 16* | 4 | 64* | 128* | 0.25 | 256* |
| 57A | 256* | 32* | 16* | 128* | 256* | 32* | 0.25 | 512* |
| 90A | 128* | 16 | 4* | 8* | 512* | 128* | 0.25 | 512* |
| 95A | 32* | 32* | 8* | 32* | 256* | 8* | 0.25 | 128* |
| 112A | 32* | 32* | 4* | 4 | 256* | 64* | 0.25 | 32* |
| 117A | 32* | 16 | 4* | 8* | 512* | 128* | 0.5* | 128* |
| 126A | 128* | 16 | 32* | 64* | 512* | 8* | 0.5* | 16* |
| 137A | 256* | 8 | 32* | 32* | 256* | 16* | 0.25 | 128* |
| **Resistance percent (%)** | 100% | 50% | 100% | 75% | 100% | 100% | 25% | 100% |

The breakpoints were obtained from CLSI except tigecycline breakpoint obtained from EUCAST. The resistant isolates are denoted by asterisk.
