# Supplementary material for: Phenotypic and genotypic characterization of linezolid resistance and the effect of antibiotic combinations on methicillin-resistant Staphylococcus aureus clinical isolates
Source: Ann Clin Microbiol Antimicrob. 2023 Apr 3;22:23. doi: 10.1186/s12941-023-00574-2 (PMC10069030; doi:10.1186/s12941-023-00574-2)
Supplement: Supplementary file 4 — Additional file 4: Table S4. Checkerboard assay results for the combinations of linezolid with other antimicrobials against LR-MRSA isolates (n=8). [file 12941_2023_574_MOESM4_ESM.docx]

**Table S4.** Checkerboard assay results for the combinations of linezolid with other antimicrobials against LR-MRSA isolates (n=8).

| **FICIs (Interpretation)** | | | | | | **Isolate Code** |
| --- | --- | --- | --- | --- | --- | --- |
| **LZD - CIP** | **LZD -TGC** | **LZD - VAN** | **LZD - ERY** | **LZD - GEN** | **LZD - CHL** |  |
| 0.75 (I) | 0.65 (I) | 0.19 (S) | 0.14 (S) | 0.39 (S) | 0.32 (S) | 9A |
| 0.19 (S) | 0.59 (I) | 2 (I) | 1.0625 (I) | 1.007 (I) | 1.04 (I) | 57A |
| 0.27 (S) | 1.003 (I) | 0.75 (I) | 0.3 (S) | 0.25 (S) | 0.25 (S) | 90A |
| 0.06 (S) | 0.635 (I) | 0.28 (S) | 0.25 (S) | 0.5 (I) | 0.43 (S) | 95A |
| 0.48 (S) | 3.21 (I) | 0.12 (S) | 0.19 (S) | 1.008 (I) | 1.016 (I) | 112A |
| 1.25 (I) | 0.907 (I) | 1.02 (I) | 0.32 (S) | 0.39 (S) | 0.75 (I) | 117A |
| 0.75 (I) | 1.76 (I) | 0.53 (I) | 1.32 (I) | 1.03 (I) | 0.45 (S) | 126A |
| 0.2 (S) | 0.84 (I) | 1.05 (I) | 0.59 (I) | 0.625 (I) | 0.15 (S) | 137A |

The FICI values were categorized into synergistic and indifferent effect, denoted by (S) and (I), respectively.
